# A Case Report and Review of Diagnostic and Therapeutic Challenges of a Malignant Peripheral Nerve Sheath Tumor in the Foot

**DOI:** 10.7759/cureus.65669

**Published:** 2024-07-29

**Authors:** Khushbu Vaidya, Raju K Shinde, Somya Goel, Kavisha Shah

**Affiliations:** 1 Department of General Surgery, Jawaharlal Nehru Medical College, Datta Meghe Institute of Higher Education and Research, Wardha, IND; 2 Department of Pathology, Geetanjali Medical College and Hospital, Udaipur, IND

**Keywords:** nerve sheath tumors, progressive swelling, discoloration of the foot, foot tumors, malignant tumors

## Abstract

Malignant peripheral nerve sheath tumors are rare. It is a soft tissue carcinoma with an aggressive nature, and it is usually associated with a poor prognosis. Common clinical presentations may include local pain, weakness, a growing mass, tingling, and numbness due to the compression of adjacent nerves or tissues, as well as weakness in affected nerves. Radiological assessment tools, such as magnetic resonance imaging and histopathological analysis, can confirm the diagnosis of malignant peripheral nerve sheath tumors. A multidisciplinary approach can manage these tumors with a timely follow-up to improve the outcomes. This is the case report of a 56-year-old female with a painless lesion on her left foot for 15 years, which recently started causing pain and a sticky discharge over the past six months. The patient was later diagnosed with a malignant peripheral nerve sheath tumor on histopathological examination, which was managed by forefoot excision without any complications. The patient recovered well at the three-month follow-up with no new complaints.

## Introduction

Malignant peripheral nerve sheath tumors are rare tumors that develop from the lining of peripheral nerves. These tumors are commonly noted with slow progression and a potential to become malignant [1]. These tumors are rare, with a reported incidence of 3%-10% of total soft tissue sarcomas. They are usually observed as a painless mass initially. These tumors can develop either de novo or from preexisting nerve sheath tumors, such as neurofibromas, particularly in patients diagnosed with neurofibromatosis type 1 (NF-1) disorder [2]. These tumors can be differentiated from other spindle-cell tumors based on immunostaining techniques such as actin, desmin, cytokeratin, and vimentin. It is found positive for S-100 antigen [2,3]. This is a case report of a 56-year-old female with a painless lesion in the left foot and discoloration of the skin for 15 years. She was managed surgically by forefoot amputation.

## Case presentation

**Figure 1:**
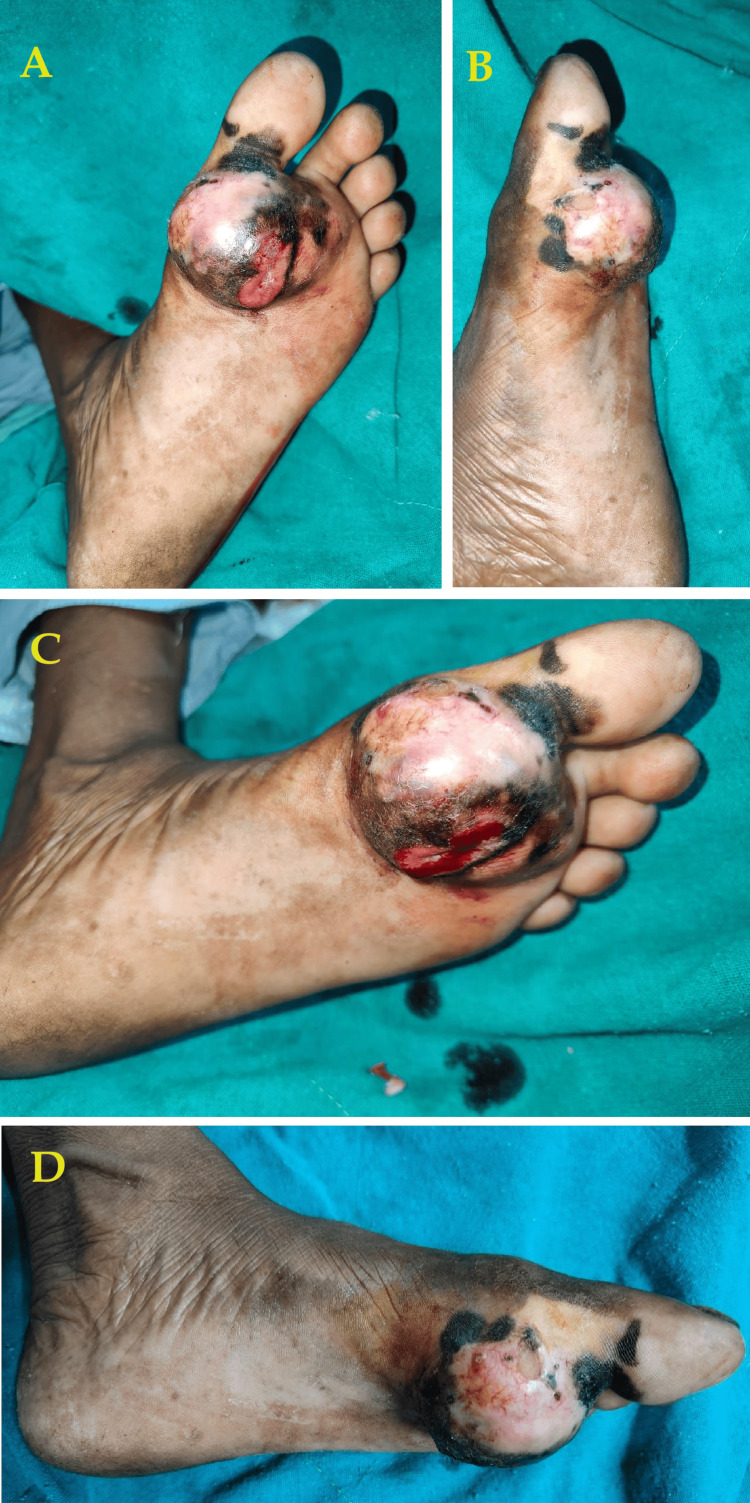
(A-D) Physical presentation of the swelling of the foot

A 56-year-old female visited the outpatient department of our hospital with a major complaint of pricking pain, swelling in her left foot, and sticky whitish discharge from the blackish-pigmented area. Self-reported history indicates that she was healthy 15 years ago but noticed a blackish swelling over the dorsum of her left foot. There was no history of any associated morbidities such as diabetes, hypertension, and addiction. The initial size observed was 0.5 × 0.5 cm, exhibiting insidious onset and gradual progression, ultimately attaining the current size of 3 × 3 cm. Similar complaints were noted over the past six months. She underwent ultrasound imaging, which revealed a well-defined hyperechoic lesion measuring 39 × 30 × 25 cm in the palmer aspect of the left foot, suggesting a probable benign lesion with potential for malignant transformation. Further advice included histopathological analysis. Excision biopsy of the same was suggestive of compound nevus. She has been receiving conservative management at a private hospital up to the present time. Physical examination revealed a swelling of approximately 3 × 3 cm with skin discoloration and black pigmentation (Figure 1).

**Figure 2:**
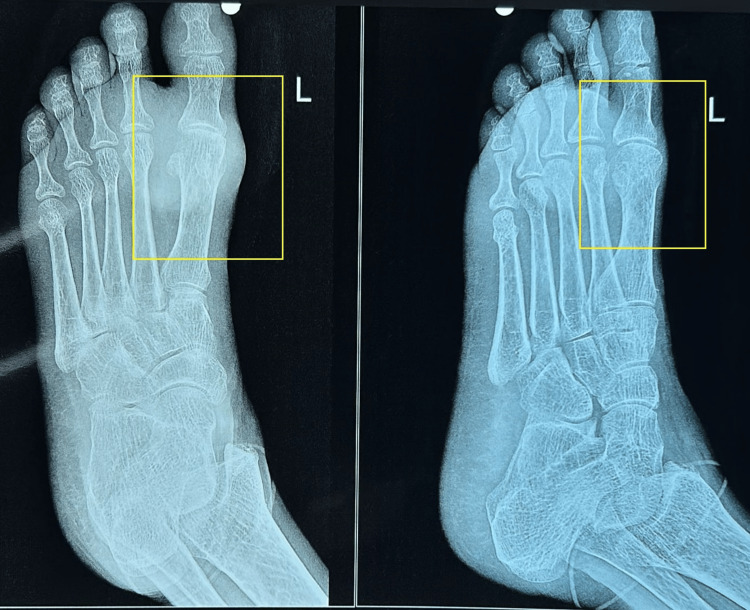
X-ray imaging of the left foot over the dorsal aspect and anteroposterior plane

The patient was subjected to X-ray radiological screening (Figure 2).

**Figure 3:**
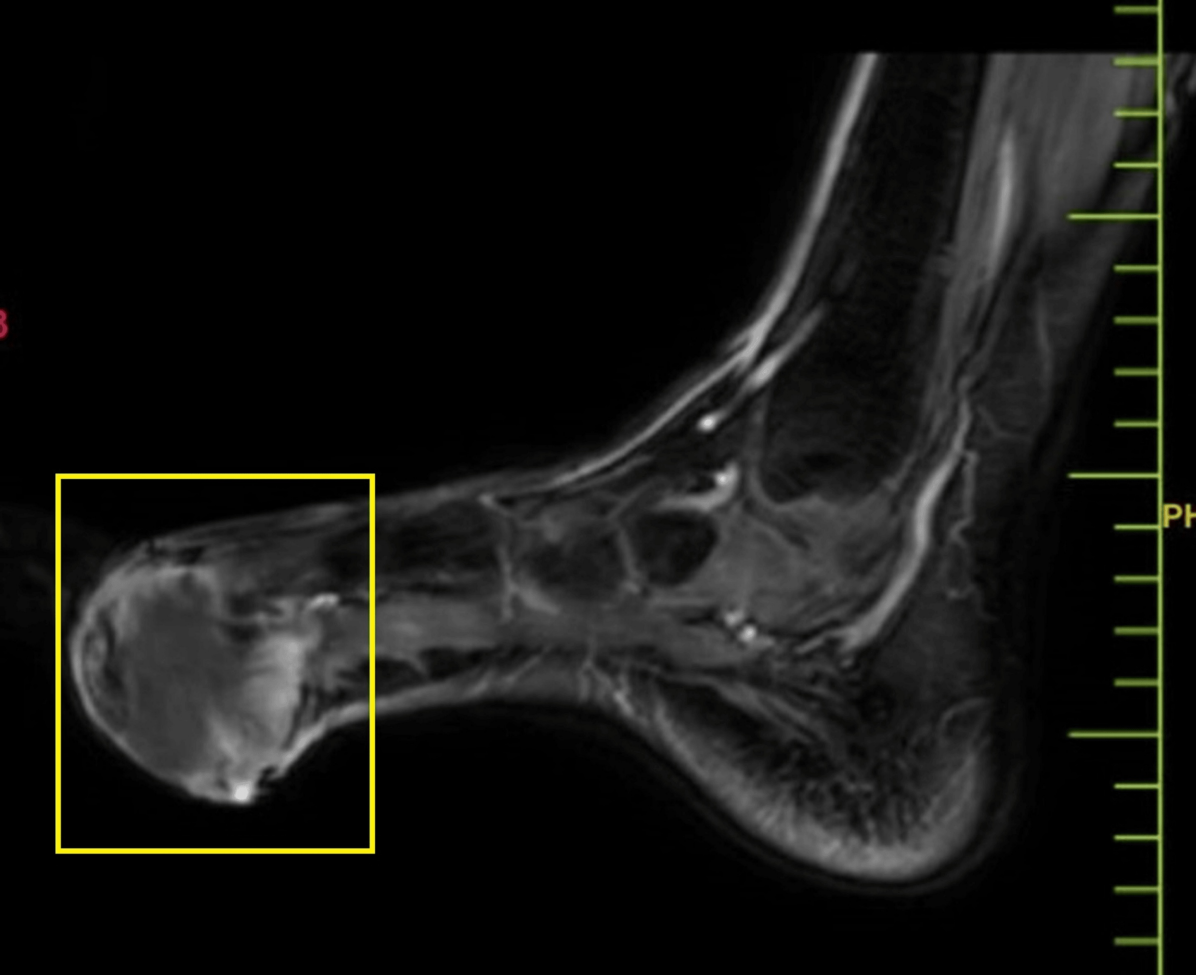
MRI of the foot (sagittal plane) MRI: magnetic resonance imaging

**Figure 4:**
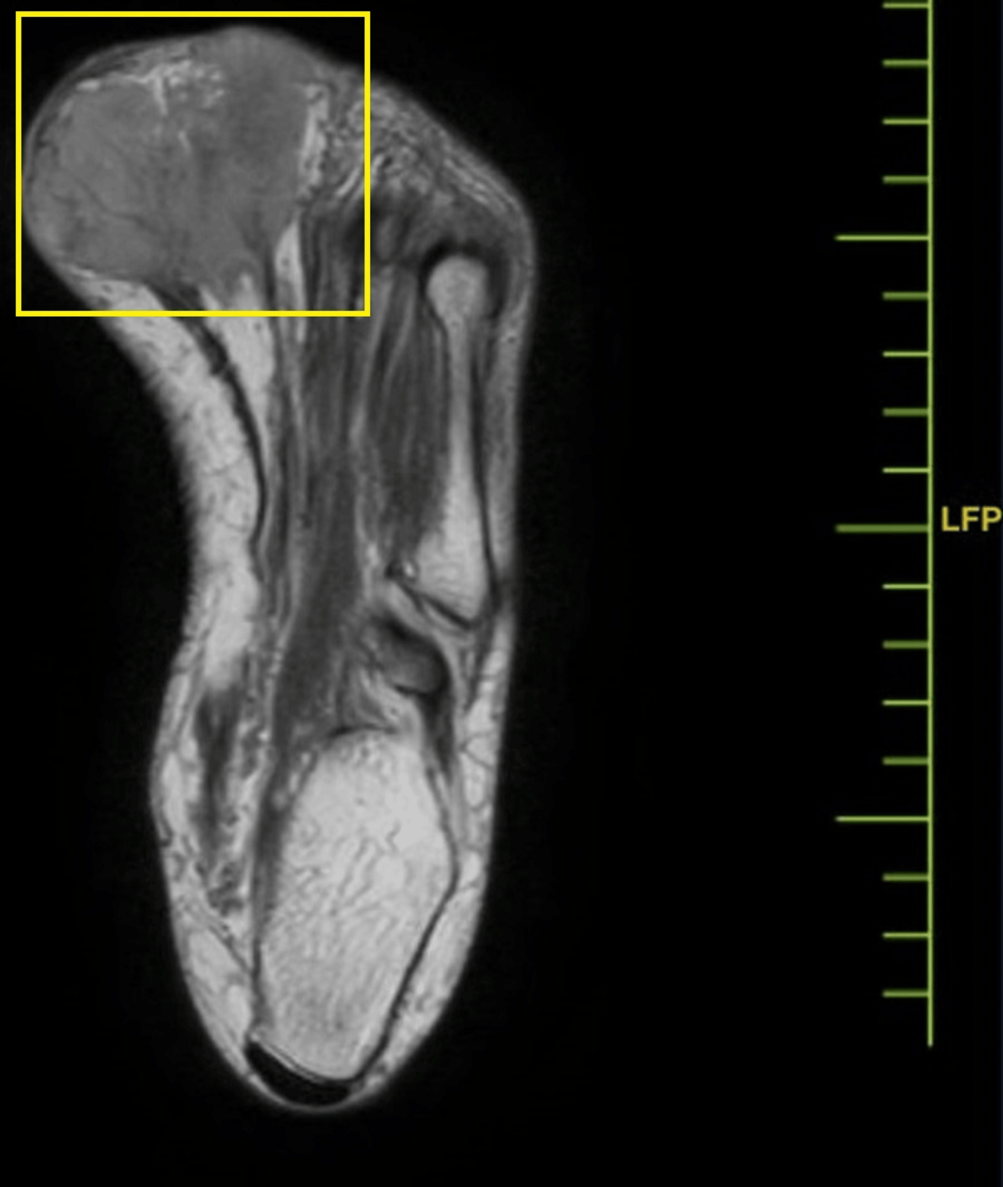
MRI of the foot (coronal plane) MRI: magnetic resonance imaging

**Figure 5:**
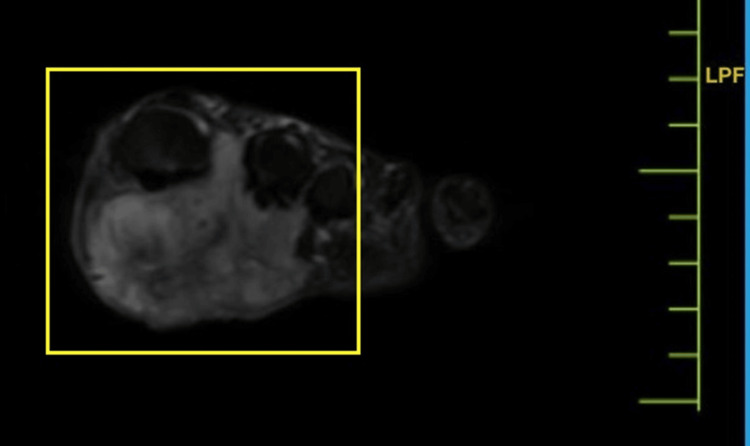
MRI of the foot (axial plane) MRI: magnetic resonance imaging

The disease severity was further assessed by magnetic resonance imaging (MRI), which indicated a well-defined lesion with altered signal intensity in the cutaneous and subcutaneous planes in the planter aspect of the foot, in the region of the first and second metatarsophalangeal joints, with benign characteristics and etiologies. MRI findings indicated a well-defined, altered signal intensity lesion in the cutaneous and subcutaneous planes of the plantar aspect of the foot, particularly in the area of the first and second metatarsophalangeal joint. The lesion extends superiorly into the first interphalangeal space. It presents as hypointense on T1-weighted imaging (T1WI), heterogeneously hyperintense on T2-weighted imaging/proton density fat-saturated, and exhibits peripheral enhancement on contrast-enhanced T1WI in the sagittal plane, coronal plane, and axial plane, respectively, in Figures 3-5. The lesion measures approximately 3 × 4.6 × 2.5 cm and was observed encasing the tendons of the flexor halluces longus and digitorum longus without any involvement of adjacent muscles and bones.

**Figure 6:**
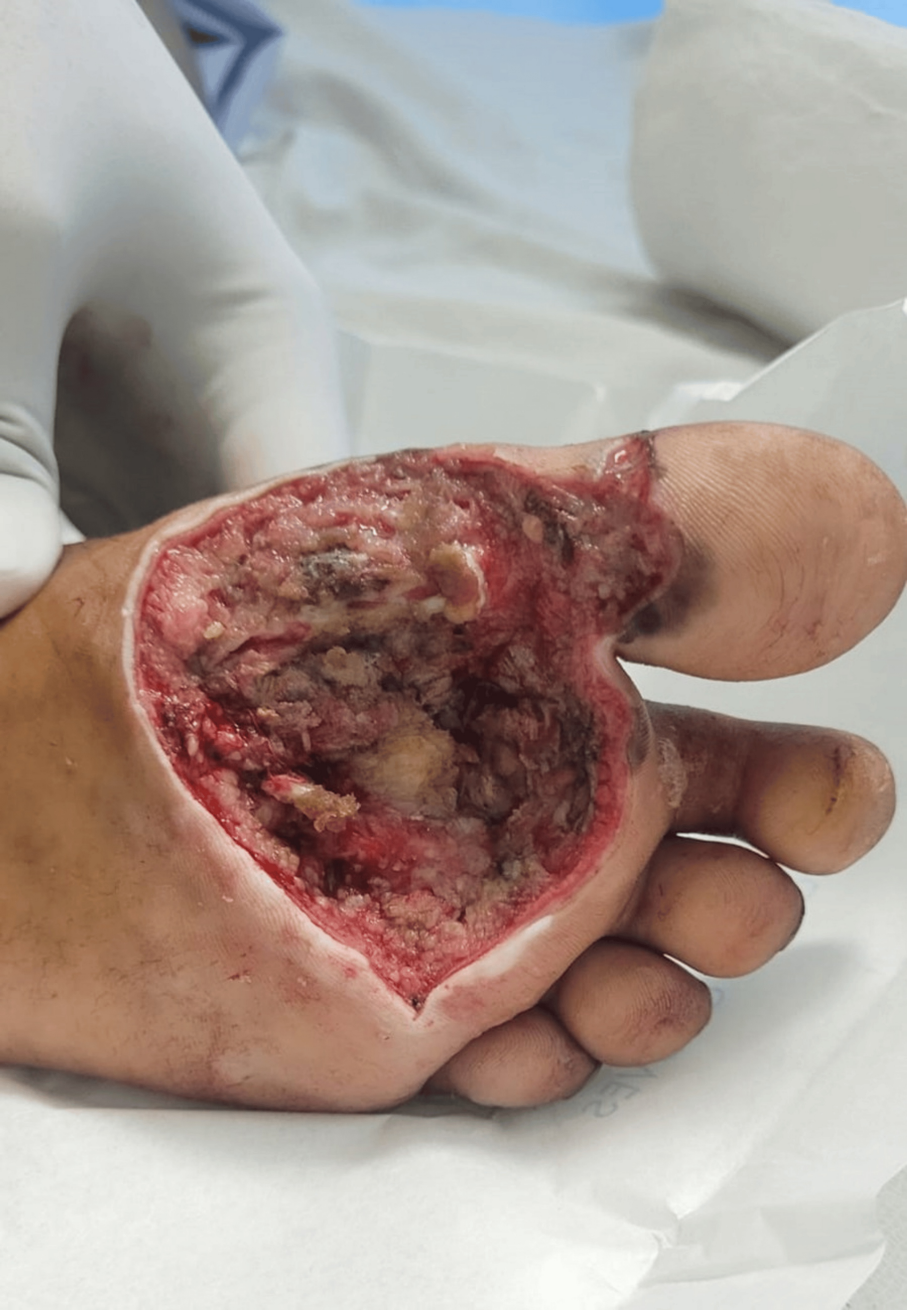
Image after tumor excision

Color Doppler was performed, revealing a few inguinal lymph nodes. The largest measured 20.4 × 7.2 millimeters on the left and 17.5 × 4.4 millimeters on the right, both with intact fatty hilum. No lymphadenopathy was noted in the bilateral popliteal region. The cervical lymph nodes were observed as follows: right submandibular 12.2 × 7.4 mm (intact hilum) with a few subcentimetric lymph nodes in the upper jugular region and left submandibular 9.9 × 7 mm (intact hilum) with a few subcentimetric lymph nodes in the upper jugular region. All other routine lab investigations were done and found to be within the normal limits. A review MRI was done, which revealed an altered signal intensity lesion in the cutaneous and subcutaneous planes of the first and second metatarsophalangeal joints. This lesion encases the tendons of the flexor hallucis longus and digitorum longus but does not affect the underlying bone. Fine needle aspiration cytology was not carried out because the samples were processed for tru-cut biopsy. A biopsy sample was taken from the site of swelling under local anesthesia and sent for histopathological examination. Histopathological examination showed a benign bone tumor known as chondromyxoid fibroma. The patient was further planned for a complete excision to rule out any suspicious sarcoma. She was evaluated for surgical fitness and underwent surgical excision. The patient underwent wide local excision under a subarachnoid block (Figure 6).

**Figure 7:**
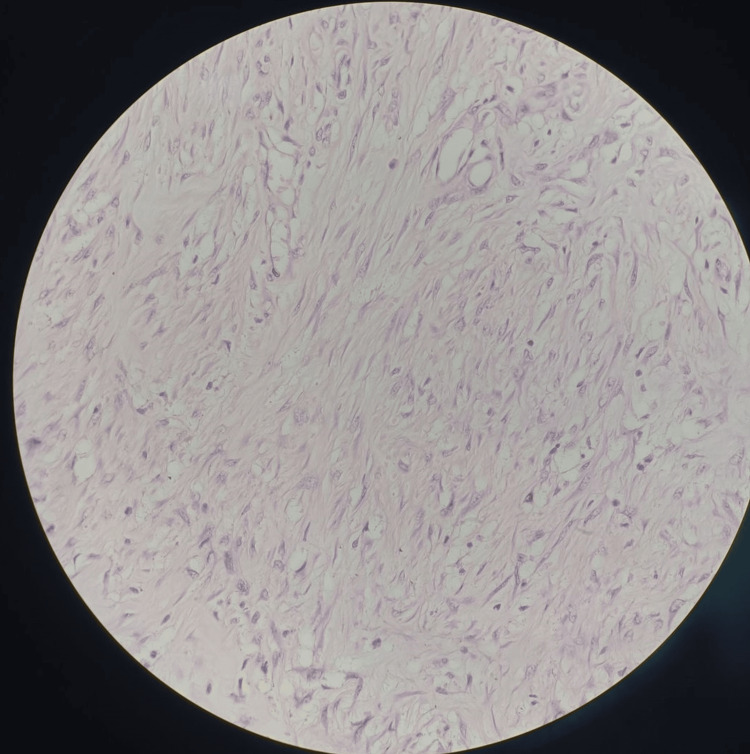
Histopathological examination of the slide suggestive of malignant peripheral nerve sheath tumor (10x magnification)

**Figure 8:**
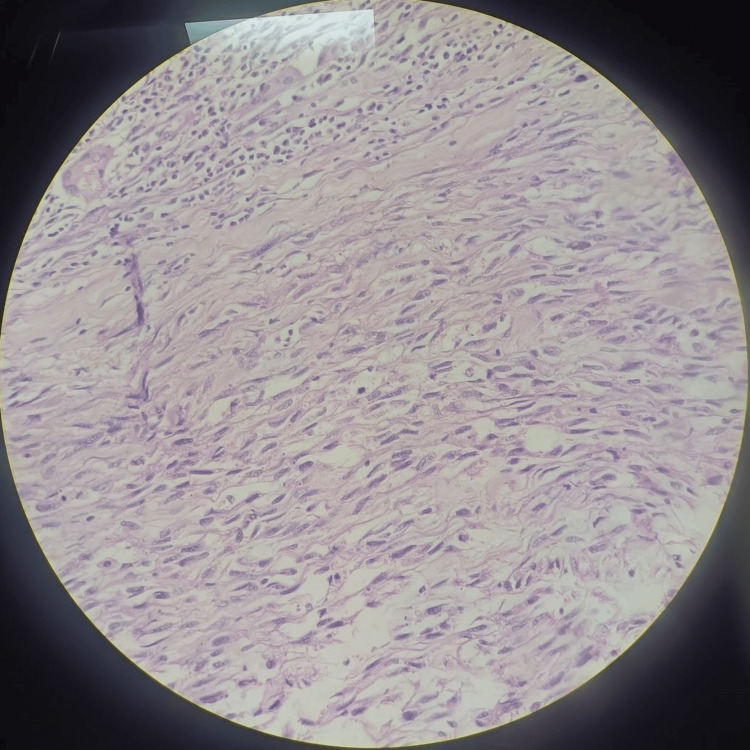
Histopathological examination of the slide suggestive of malignant peripheral nerve sheath tumor (40x magnification)

The procedure was uneventful, and the excised specimen from the planter aspect was sent for histopathological examination. The findings revealed the presence of a malignant peripheral nerve sheath tumor with a surrounding positive margin and a chondrosarcomatous component (Figures 7, 8).

**Figure 9:**
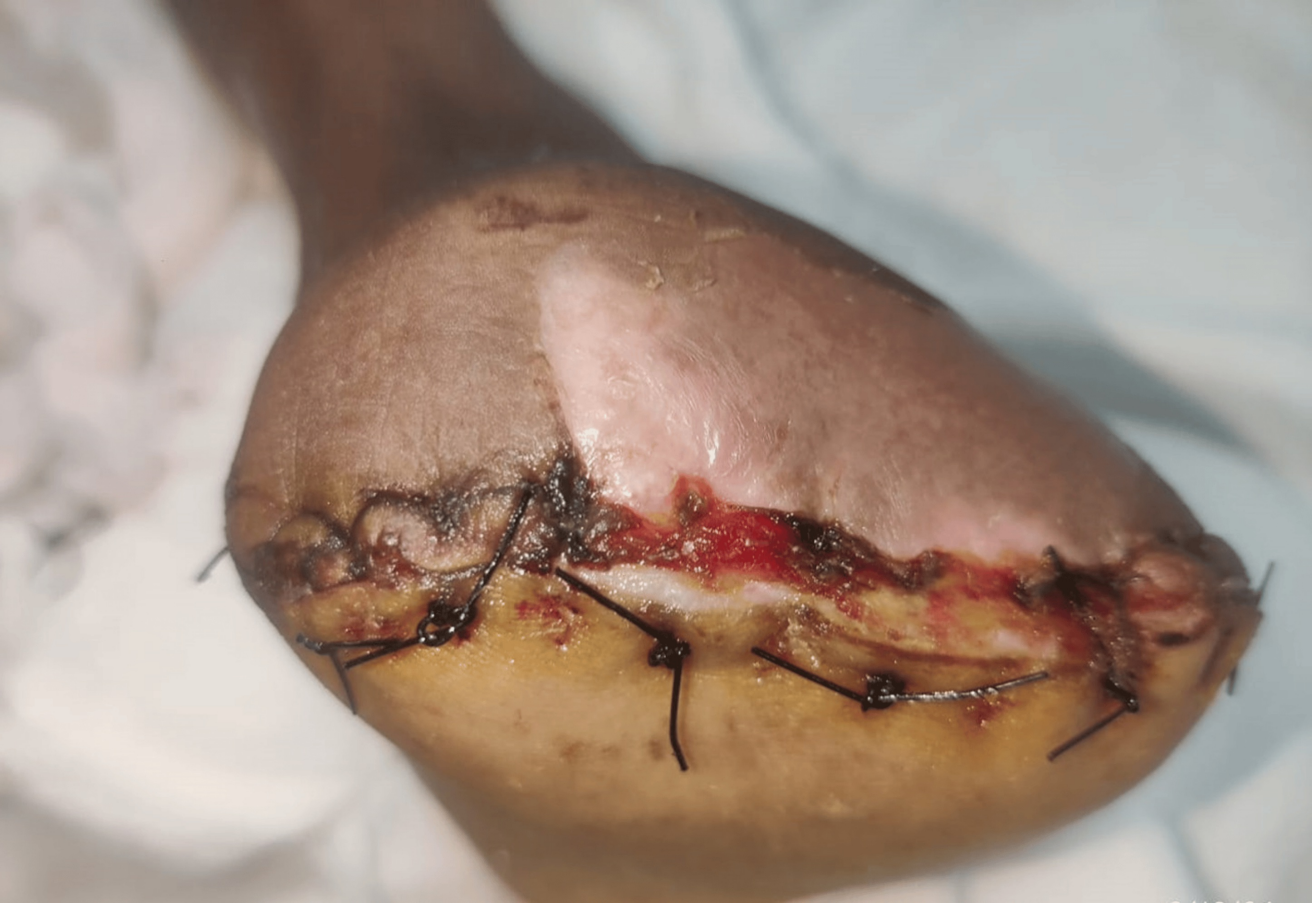
Postsurgery forefoot amputation image

The immunohistochemistry report indicated that S-100 was positive and KI67 was greater than 80%. After surgery, the patient received routine postoperative antibiotics, antacids, analgesics, and multivitamins, along with necessary dressings and physiotherapy. Follow-up USG showed a few subcentimetric inguinal lymph nodes in a bilateral inguinal region measuring 9.1 × 3.5 millimeters on the right and 8.1 × 4.1 millimeters on the left with maintained hilum. A decision was made to revise the margin study and perform a Chopart amputation, and the patient was scheduled for a left forefoot amputation. The procedure was completed after necessary fitness approvals, and the patient underwent Lisfranc amputation of the left foot under unilateral spinal anesthesia with an uneventful procedure (Figure 9). The patient was discharged 15 days postprocedure with a follow-up in the surgery outpatient department every three months until one year.

The patient was discharged 15 days postprocedure with a follow-up in the surgery outpatient department every three months until one year.

## Discussion

Malignant peripheral nerve sheath tumors are also termed neurofibrosarcoma, neurogenic sarcoma, malignant schwannoma, and malignant neurilemmoma. These tumors are common in the upper and lower extremities in adults between 20 and 50 years of age. Common presentation sites commonly include the head, neck, and upper body. However, research literature indicates that tumors can also occur in the lower parts of the body [4]. These masses are commonly seen under the skin, exhibiting gradual enlargement. They are often associated with a weakening of the associated nerve function and problems with the normal functioning of the related body part [2,5]. Malignant peripheral nerve sheath tumors are reported to be associated with NF-1 gene mutation, and its incidence in both benign and malignant forms is associated with NF-1 genetic mutation. The NF-1 gene is a tumor-suppressing gene that is present on chromosome 17, encoding neurofibromin protein [4-6]. Radiation exposure is also reported as a major risk factor for these tumors apart from the genetic associations, with 10% of the total cases exposed to radiation. Around 50% of the cases had a positive NF-1 [7]. NF-1 has also been linked to the formation of plexiform neurofibromas from the Schwann cells, leading to the loss of its heterozygosity [8]. Pain is a common symptom associated with these lesions or tumors, but that was not the case in our patient. She had a lesion for the past 15 years, and the history of the associated pain was only from the last six months. Also, malignant peripheral nerve sheath tumors are known for their aggressive growth, which was also found to be in contradiction in this case, as the patient reported the lesion to be present for the past 15 years without any associated pain or discomfort. These tumors are associated with local recurrence and metastatic transformation; hence, surgical resection with a multimodal approach is recommended [9]. It can be summarized that malignant peripheral nerve sheath tumors pose significant treatment challenges attributed to their aggressive nature. An improved understanding of their molecular drivers may lead to better targeted therapies in the future.

## Conclusions

In conclusion, timely diagnosis and intervention can improve outcomes for malignant nerve sheath tumors of the foot. Prompt intervention can help prevent malignant transformation and metastasis, as malignant nerve sheath tumors may appear benign upon primary presentation. Surgical resection and adjuvant therapy are recommended to prevent recurrence and adverse outcomes.
